# Regional Differences in Intestinal Parasitic Infections among Army Recruits in a Southern Mozambique Training Center: A Cross-Sectional Study

**DOI:** 10.3390/pathogens12091105

**Published:** 2023-08-29

**Authors:** Verónica Casmo, Sérgio Chicumbe, Rosa Chambisse, Rassul Nalá

**Affiliations:** 1 Instituto Nacional de Saúde (INS) EN1, Bairro da Vila-Parcela n 3943, Distrito de Marracuene, Maputo C.P. 264, Mozambique; chicumbe@gmail.com (S.C.); rassulmn@gmail.com (R.N.); 2 Department of Cell and Molecular Biology, Institute of Cell and Microbiology, Uppsala University, 76124 Uppsala, Sweden; 3 National Directorate of Military Health, Maputo C.P. 3216, Mozambique; rosaguambe@yahoo.com.br

**Keywords:** intestinal parasites, army, recruits, Mozambique

## Abstract

Due to the high prevalence and diversity of clinical manifestations, intestinal parasitic infections (IPIs) represent a public health problem. The objective of the work was to determine the prevalence of IPIs among army recruits at a practice and training center in southern Mozambique. Sociodemographic information was obtained through semi-structured interviews. Single urine and stool samples were collected from 362 recruits. Parasite diagnosis was made by filtration, formaldehyde-ether and Kato-Katz techniques. Positive individuals underwent abdominal ultrasound. Then, descriptive statistics and cross-tabulations were performed, and *p*-values < 0.05 were considered significant. The prevalence of infection with at least one parasite was 25.1% (95% CI: 20.5–29.6; n = 91). The most common parasites were *Entamoeba coli* (10.7%; 95% CI: 7.4–13.7; n = 37) and *Trichuris trichiura* (6.1%; 95% CI: 4.6–9.9; n = 25). Parasitic infection was associated with the origin of the participant (*p*-value < 0.001), and the province of Sofala had the highest prevalence among the provinces studied (70.6%; 95% CI: 47.0–87.8; 12/17). Since oral fecal transmission occurs for several parasites, routine screening and deworming prior to enrollment at the army training center is recommended to reduce transmission of intestinal parasites among recruits.

## 1. Introduction

Parasitic infections are a major public health problem, especially in low-income countries [1]. The World Health Organization (WHO) indicates that helminth and protozoan infections affect about 3.5 billion people, with 450 million people developing diseases associated with intestinal parasites worldwide [2]. Major health problems caused by intestinal parasites include the negative effects on nutritional status, physical growth and cognitive development, especially in children.

Among all infections, those caused by helminths and protozoa stand out with the highest prevalence rates and are responsible for significant morbidity, especially in low-income countries. While the incidence of these infections is approximately 50% in developed countries, in underdeveloped countries, the incidence can reach up to 95% [3].

The socioeconomic status of a population is closely related to the prevalence of intestinal parasitic infections within that population [4]. It is known that intestinal parasitic infections are related to several factors such as inadequate sanitary facilities, contaminated water and food, sociocultural factors, contact with animal and human feces and lack of basic hygiene. In addition, the characteristics of a parasite, such as developmental stage and species, may contribute to higher parasite prevalence [5].

Another factor that must be considered when evaluating parasite prevalence is age. Most of the studies related to intestinal parasitic infections carried out in Mozambique have focused on children of preschool [6] and primary school age [7,8,9,10,11], since these age groups have higher parasitic prevalence compared to adults [12,13].

Determining intestinal parasite prevalence and distribution is a key step in establishing effective control programs, with the ultimate goal of improving population health [14]. Recently, efforts were made by Mozambican health authorities to reduce intestinal parasite prevalence through the regular administration of deworming medicines to children. However, combating parasites requires multisectoral actions, which not only focus on the treatment of at-risk populations, but also on disease prevention.

The recruitment process for military service in Mozambique does not routinely involve screening for parasites in feces and urine. This population would be important to screen, however, as military personnel are recruited from a range of urban, peri-urban, or rural settings, many of which are ecologically conducive to a high prevalence of intestinal and bladder parasites.

This study aimed to determine the frequency of intestinal and bladder parasites among recruits residing at an army practice school in Mozambique and explore the sociodemographic characteristics associated with parasitic infection. The results of this study can help the intestinal parasite infection control program, with the purpose of improving the health of the population.

## 2. Results

Urine and fecal samples were collected from a total of 362 military recruits residing at an army practice school in southern Mozambique. Recruits were primarily between the ages of 20 and 27 years (n = 271; 74.9%). During the participant interview process, some recruits did not provide sociodemographic data (missing data for age: n = 4); province of origin: n = 2; education level: n = 9; occupation: n = 71; and religion: n = 59 (Table 1).

**Figure 1:**
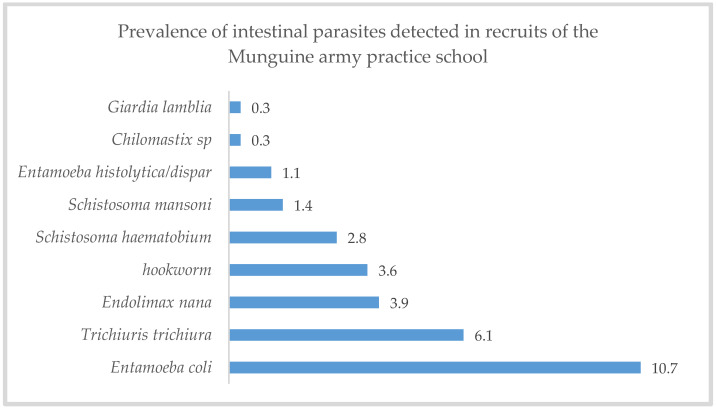
Prevalence of intestinal parasites in recruits of Munguine army practice school, Maputo-Mozambique (N = 362).

From the analysis of the biological material collected, parasitic infection of at least one parasite was observed in 25.1% (95% CI: 20.5–29.6; n = 91) and mixed infection was observed in 7.2 % (95% CI: 4.9–10.2; n = 26) of recruits. The most common parasites were *Entamoeba coli* (10.7%; 95% CI: 7.5–14; n = 39) and *Trichuris trichiura* (6.1%; 95% CI: 3.6–8.6; n = 22). *Giardia lamblia* and *Chilomastix* were the least common parasites (0.3%; 95% CI: 0.3–0.0.8; n = 1, each) (Figure 1). An intensity of *Schistosoma haematobium* eggs was found, which varied between 1 to 36/10 mL of urine, and about ten recruits were infected.

**Figure 2:**
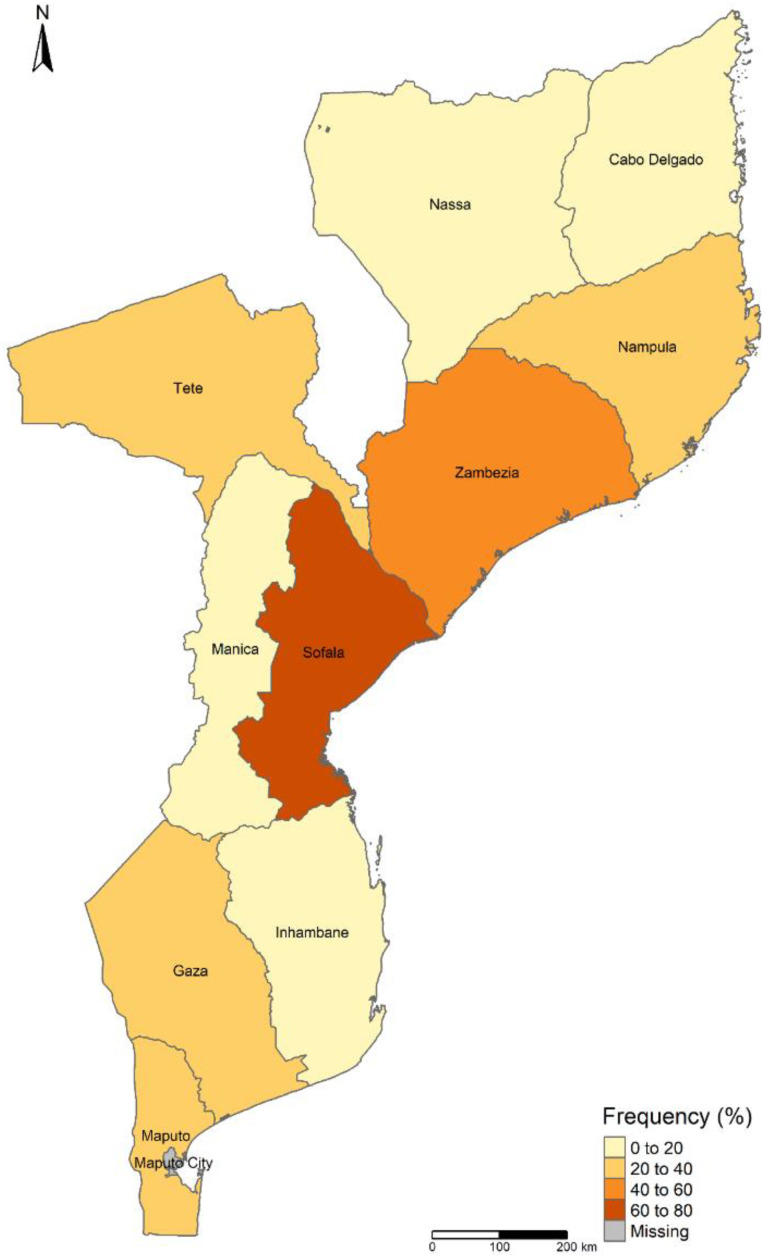
Distribution of intestinal parasites detected by provenance among recruits of the Munguine Army Practice School in southern Mozambique.

Recruits from Sofala province had the highest prevalence of parasitic infection, Figure 2, (70.6%; 95% CI: 47.0–87.8; 12/17), while Niassa province had the lowest (13.3%; 95% CI: 4.7–28; 74/30) (Table 1), among the provinces evaluated in this study. Gender, age group, education, occupation and religion were not significantly associated with parasitic infections (*p* > 0.05).

From the abdominal ultrasounds performed on recruits who had positive results (n = 91), abnormalities were observed in five participants, such as bladder, ureteral and renal changes (Table 2).

## 3. Discussion

To the best of our knowledge, this is the first study to assess the prevalence of parasites among army recruits in Mozambique. In this work, high levels of helminth and soil-transmitted protozoan infections were observed, including infections by multiple parasitic species in some recruits.

This study also demonstrated a high prevalence of parasitic infections among adults between the ages of 20 and 27, whereas most previous studies on parasitic infections in Mozambique focused on younger populations. Though no significant difference in infection prevalence was identified between men and women, it is possible that this result was impacted by the unequal proportions of male and female participants (75% versus 25%) in the study group. These unequal proportions were expected, as, historically, the Mozambican army has been mostly composed of men. However, a previous hospital-based study conducted in Southern Mozambique showed lower rates of infections in females compared to males of the same age group [13].

Among the parasites identified, *Entamoeba coli* was the most common (10.7%), a result similar to that was found in other studies about intestinal parasites with HIV-infected and -uninfected patients in Maputo-Mozambique and in intestinal parasite-infected patients in Kwazulu Natal, South Africa, respectively [15,16]. However, the prevalence of *E. coli* was significantly lower than that in a study carried out in the province of Nampula, Mozambique, which demonstrated an *E. coli* infection prevalence of 51% [10]. Although *E. coli* was the parasite most frequently detected in the present study, it is part of a group of non-pathogenic parasites. However, its presence is still a strong indication of poor hygienic conditions and co-infection with other pathogens [14].

Other intestinal parasites also stood out in this study, namely *T. trichiura*, *A. lumbricoides* and *hookworms*. These parasites are commonly detected in studies related to intestinal parasites in Mozambique [9,10,11,17]. According to the WHO, Mozambique had about 12 million children in need of treatment for soil-transmitted helminths, including *T. trichiura*, *A. lumbricoides* and *hookworm*.

In addition to single infections, mixed or multiple infections were observed in recruits, similar to the results found by other researchers [10,15]. Co-infection is common in environments with high levels of parasitic contamination [18]. The multiparasitism found in this study likely indicates that a high percentage of the participants were exposed to an environment contaminated by different parasitic species. The most common co-infections were *E. coli* and *E. nana*, as well as *A. lumbricoides* and *T. trichiura*. This finding aligned with the results of other parasite co-infection studies carried out in Mozambique [10]. Both *E. coli* and *E. nana* are non-pathogenic parasites whose presence in the human body is justified by exposure to contaminated environments as well as lack of hygiene. *A. lumbricoides* and *T. trichiura* are parasites that live the same environment, also justifying their co-occurrence in some recruits [9].

According to Augusto et al. [7,8,9,10,11], intestinal parasites are widespread in Mozambique, but with different infection rates from one place to another. It is important to point out that the province of Sofala, unlike the rest of Mozambique, is characterized by having a water table very close to the surface of the earth, which makes the soil prone to flooding. This, together with the lack of sanitation, characteristic of this province, may have contributed to the high prevalence of intestinal parasites among Sofala recruits. As for recruits from Niassa province, a relatively low prevalence of intestinal parasites was observed, which may be related to the province being characterized by having dry soil with a deep-water table and environmental conditions that limit transmission, such as low population density.

Abdominal ultrasounds performed in individuals positive for intestinal parasites showed normal ultrasound results in almost the entire population, where only 5 of 91 recruits showed abnormalities, but without direct clinical association between the observed abnormality and the occurrence of intestinal parasites, except for ultraviolet signs and sonographic scans of hematuria (urinary scattered hyperechogenic spots). The small number of abnormalities detected may, however, indicate that abdominal ultrasound may not be a sensitive tool to detect minor intestinal morbidity by *Schistosoma* sp. and other parasites, a conclusion drawn in a study conducted in Niamey, Niger [19]. Relatedly a study carried out in Italy concluded that intestinal ultrasound may not be a useful tool in all scenarios for a more comprehensive assessment of morbidity before and after treatment of intestinal infection [20]. This result can be explained by the fact that the structural abnormality of parasitic infections are long-term changes.

According to Knopp et al. [20] and WHO definitions, schistosomiasis with a load of 1 to 25 eggs per 10 mL of urine is considered a mild infection, 25 to 50 eggs per 10 mL of urine is moderate and a severe infection is indicated by more than 50 eggs per 10 mL of urine [20]. Although this study recorded an intensity that ranged from 1 to 36 eggs per 10 mL of urine, the moderate intensity was observed in only one individual, and only 2.8% (n = 10) of the individuals had schistosomiasis. Screening for parasites using only a urinary or intestinal sample for parasites may have influenced the results, since the production and/or elimination of eggs of these parasites is not regular [21].

## 4. Materials and Methods

### 4.1. Study Period and Area

A cross-sectional study was conducted between July and August 2016 at a military training school in southern Mozambique. The school, which offers course durations of at least three months, is located about 60 km from Maputo city, the capital of Mozambique. One year before our study, there were about 1200 recruits at the school.

### 4.2. Enrollment Process

Prior to enrollment, a sample size was estimated considering an overall population of 1200, an assumed prevalence of 50% for intestinal parasites, a desired precision estimate of 5% and a confidence interval of 95%, which resulted in a minimum sample size of 292. Simple random sampling was used to identify participants. According to Lwanga and Lemeshow a prevalence of 50% of intestinal parasites in recruits was estimated due to lack of data from similar studies in Mozambique [22].

For justified reasons, the number of recruits in the school was not provided. For this, 1200 was assumed, which was the number of recruits in the school one year before the study.

Researchers engaged management at the school to obtain voluntary consent from recruits, accounting for the fact that there is an inherent vulnerability of the recruits to hierarchical decisions. The purpose of the study was explained to all the recruits, including the need to voluntarily consent to their participation and the ethical principles of human research participation and rights they were entitled to. Written informed consent was obtained from all participants.

### 4.3. Data Collection and Analysis

A semi-structured interview was provided to collect sociodemographic data from the selected recruits. Two sterile vials, one to collect urine and one to collect stool, were given to each participant. The recruits were instructed to collect the urine samples after physical exercise. The samples and questionnaires were collected the following morning after enrolment. Specimens were transported in thermal boxes to the Instituto Nacional de Saúde (INS) reference parasitology laboratory and processed using Ritchie’s method for stool (1948) [23], which is based on a methodology recognized for its efficacy diagnosing helminths and protozoa, the Kato-Katz laboratory method for stool adopted by the WHO as the gold standard for the diagnosis of human *Schistosoma mansoni* infection as described by Katz et al. [24] and urine filtration, a quantitative, simple, low-cost method recommended for research of *Schistosoma haematobium* eggs in urine by the WHO [25].

All participants with any positive result for helminth eggs (stool sample) and/or positive result for *Schistosoma haematobium* eggs (urine sample) were dewormed with Albendazol and Praziquantel tablets, as per the national protocol. A positive result for any parasite received an abdominal ultrasound examination. All recruits reported, in the questionnaire, not having taken any deworming agents in the last six months before the study. It should be noted that in Mozambique, mass deworming is not a common practice in adults. To minimize errors, data were double entered in a Microsoft Excel spread sheet and analyzed in R version 4.1.0. Univariate and cross-tabulation between sociodemographic and intestinal parasites was made, including estimates of confidence intervals (CI) for the proportions. A *p*-value of < 0.05 was considered significant. Given the nature of the army environment, parasitological analyses were performed with only one sample from each recruit, instead of three, as is often recommended.

## 5. Conclusions

There is a high prevalence of parasitic infections among army recruits in Munguine, with 10.7% for *E. coli* and 6.1% for *T. trichura*. The provenance of recruits was the notable factor found to be associated with parasitic infection, with recruits from Sofala province having the highest prevalence of parasitic infection and those from Niassa province having the lowest prevalence. Routine screening before enrolment in the military training centers is recommended along with general deworming to reduce transmission of intestinal parasitic infections in endemic regions for these infections.

## Figures and Tables

**Table 1:** Frequencies of intestinal parasites by sociodemographic characteristics of Munguine military recruits (N = 362).

Characteristics	%(^c^)	n/N	*p*-Value
Gender			0.701 ^a^
Male	25.8 (20.9–31.3)	70/271	
Female	23.1 (15.3–32.5)	21/91	
Age in years (categorized)			0.270 ^a^
20–22	26.8 (21.3–32.8)	61/228	
23–24	24.3 (16.9–33.0)	26/107	
≥25	13.0 (3.87–28.7)	3/23	
Missing		4	
Provenience			<0.001 ^a^
Niassa	13.3 (4.7–28.7)	4/30	
Inhambane	14.0 (6.0–26.5)	6/43	
Cabo Delgado	16.7(5.9–34.9	4/24	
Manica	17.1 (7.5–32.0)	6/35	
Gaza	20.0 (8.1–38.4)	5/25	
Nampula	23.3 (12.6–37.3)	10/43	
Tete	24.1 (11.5–41.6)	7/29	
Maputo	28.8 (19.7–39.3)	23/80	
Zambezia	41.2 (25.9–57.9)	14/34	
Sofala	70.6 (47.0–87.8)	12/17	
Missing		2	
Education			0.884 ^a^
Basic	25.2 (20.4–30.5)	70/278	
Elementary	23.3 (12.6–37.3)	10/43	
Middle/Superior	28.1 (14.9–45.1)	9/32	
Missing		9	
Occupation			0.748 ^b^
Employed	24.6 (17.5–32.9)	29/118	
Non-employed	25.0 (7.6–52.9)	3/12	
Student	21.1 (15.4–27.9)	34/161	
Missing		71	
Religion			0.081 ^b^
Catholic	23.2 (18.1–28.9)	54/233	
Muslim	23.5 (13.6–36.4)	12/51	
Others	47.4 (26.6–68.8)	9/19	
Missing		59	

a: Pearson chi-square test; b: Fisher’s exact test; c: Jeffrey’s confidence intervals.

**Table 2:** Ultrasound results among recruits from the Munguine Army Practice School in southern Mozambique.

Kind of Alteration	Topography	Number	Sex	Parasite
Scattered heterogenicity	Bladder	2	Male	*S. haematobium*
Left Pieloectasia 1.5	Ureter	1	Male	*T. trichiura*
Focal scar	Kidney	1	Female	*T. trichiura*
Simple cyst	Kidney	1	Male	*E. coli* and *T. trichiura*

## Data Availability

The data presented in this study are available upon request made to the corresponding author.
